# Metabolomics reveals chemical changes in *Acer saccharum* sap over a maple syrup production season

**DOI:** 10.1371/journal.pone.0235787

**Published:** 2020-08-20

**Authors:** E. Jose Garcia, Tim McDowell, Cheryl Ketola, Michael Jennings, J. David Miller, Justin B. Renaud

**Affiliations:** 1 Fanshawe College, School of Applied Science and Technology, London, ON, Canada; 2 London Research and Development Centre, Agriculture and Agri-Food Canada, London, ON, Canada; 3 Department of Chemistry, Carleton University, Ottawa, ON, Canada; Luleå University of Technology, SWEDEN

## Abstract

Maple syrup, made by boiling the sap of *Acer saccharum*, is an important agriculture commodity in eastern Canada and New England. Although the collection season is relatively short, a rich progression in the sensory qualities of maple syrup can occur throughout the season. A risk associated with maple syrup production at the end of a season is the development of off-flavors that result in syrup with little to no commercial value. Maple syrup producers in Canada and the USA call this ‘buddy syrup’. In this study, sugar maple (*Acer saccharum*) sap was collected in sequential samples through the harvest season from stands across Ontario. Metabolomics analysis of the sap samples was performed by high-resolution mass spectrometry. This revealed an evolution of the chemical composition, mainly occurring 30 days prior to leaf emergence. The major chemical constituent of maple syrup, sucrose, decreased sharply in late season sap, driven by microbial activity. The alditol mannitol increased in late season sap to concentrations ≥2 mg/mL and is likely an indicator of the start of photosynthesis. Amino acids, notably methionine and asparagine were present in higher amounts in late season sap. Non-targeted analysis revealed a series of related compounds that contained quaternary ammonium moieties including choline, hercynine, trigonelline, glycine betaine and carnitine increased in late season sap. These classes of compounds could act as methyl donors during the heating/evaporation of sap into syrup, affecting taste. Based on descriptions of the nature of buddy syrup and an extensive literature on flavors in foods, the amino acids methionine and asparagine were found as likely precursors to the compounds responsible for buddy syrup.

## Introduction

Maple syrup is made by boiling the sap of sugar maple (*Acer saccharum*) collected in the early spring when the temperature is above freezing during the day and colder at night. The Indigenous peoples where sugar maple grows have produced maple syrup/sugar for generations. The methods were taught to early settlers and the product became a commercial industry in the early part of the 20^th^ century [1]. Maple syrup and other maple products are an important agricultural commodity in Quebec, New Brunswick and Ontario as well as a number of US States, notably Vermont. In 2017, Canada produced 47.3 million liters of maple syrup, amounting to a value of $C 494 million [2].

Although the main organic component of maple syrup is sucrose, sugar maple sap of *A*. *saccharum* is comprised of many organic and inorganic compounds including reducing sugars, amino acids, peptides, proteins, organic acids and phenolic compounds [3]. A unique property of maple syrup is the changes to the sensory qualities that occur throughout a short production season of usually 6 weeks or less [4]. At the onset of the season, maple syrup often has a lighter colour and delicate taste, becoming progressively darker with more intense flavors later in the season. As the tree’s buds begin to swell, syrup made from sap collected at this time has an unpleasant odour and taste, called ‘buddy’ and cannot be sold. This seasonal development in flavor is speculated to be the result of changes in the chemical composition of the sap. Sap chemistry also varies by the locations of the tree stand because of variation in soil chemistry. For example, there are significant differences in syrup pH and concentration of salts [5].

As the trees exit their winter dormancy, the concentration of amino acids in the xylem increases, with nitrogen being mobilized through the xylem for eventual leaf growth [6, 7]. Although the xylem of sugar maple is essentially sterile [8, 9], wild yeasts that grow in the transfer lines and collection vessels have an influence on the taste of the syrup [10, 11]. In part, this activity on the colour and flavor of syrup is thought to result from the inversion of sucrose to fructose and glucose [8, 9].

The chemicals responsible for the distinct maple flavor are not present in the sap itself; they result from numerous thermally driven chemical reactions during the evaporation process. Both Findlay and Snell in 1935 [12] and Holgate in 1950 [8], independently showed that sap concentrated into syrup without heat (vacuum distillation and freeze concentrating respectively) lacked a characteristic maple flavor. Conversely, heating reducing sugars and some amino acids will produce products with ‘maple-like’ flavor [13]. The production of these flavor compounds by heating is the result of thermally driven chemical processes such as Maillard reactions between reducing sugars such as fructose and glucose with amino acids.

Small changes to the chemical composition of the sap, caused by the tree itself or microbial activity have a large impact on the sensory qualities of the syrup. Not only is the tree sap concentrated 40–50 times during processing, some chemicals that are produced including sotolon (4,5-dimethyl-3-hydroxy-2(5H)-furanone) found between 0.03–0.56 ppm in syrup, have very low flavor thresholds [14, 15]. Therefore, the appearance of off-flavors in syrup may be the result of subtle alterations in the chemical composition of the sap. It has been suggested that alkyl-pyrazines, formed by heating amino acids and reducing sugars [16, 17] are a causative agent of ‘buddy’ off-flavors in syrups [18, 19]. Others have suggested that dimethyl disulfide (DMDS) and dimethyl trisulfide (DMTS) may also contribute to ‘buddy’ flavoring [20]. Although N’guyen *et al*. (2018) proposed that microbial activity plays a determining role in the development of ‘buddy’ syrup [6], Holgate showed that aseptically collected sap could also produce this off-flavor in late season [8].

To date, many studies have focused on the chemical characterization of maple syrup itself. Examining the chemical composition of maple sap has employed classical, targeted analysis whereby a suite of known analytes are detected and quantified [6, 21]. High-resolution mass spectrometry reveals information on all ionizable compounds present within a sample. Untargeted analysis applied to the analysis of maple sap, has yielded new findings, that could expand our understanding of the development of maple syrup across a production season. In this study, 282 maple sap samples were collected from 12 sites across the province of Ontario, Canada, during the 2019 maple syrup production season. A non-targeted metabolomics approach was implemented to characterize the samples. This data provides a baseline for better understanding of the chemical changes occurring within the sap across the season, and the role they may play in the sensory properties of the maple syrup that is produced, including off-flavors.

## Materials and methods

### Maple sap sampling

In 2019, maple sap samples were collected over the entire production season by 12 members of the Ontario Maple Syrup Producers Association (OMSPA). These farms covered all the OMSPA regions of Ontario (https://www.omspa.ca/). Sap samples (n = 282) were collected as approximately 10 ml aliquots into 15 ml plastic centrifuge tubes contain 1.5 mg of sodium benzoate and stored at 4 ºC. At the end of the production season the samples were shipped on ice to Agriculture Canada’s London Research and Development Centre (London, Canada) and immediately stored at 4 ºC. Samples analyzed by C18 chromatography were prepared by diluting 200μL of sap 1:1 with methanol. Sample analyzed by HILIC chromatography were prepared by diluting 200 μL of sap 1:2 with 30 mM ammonium formate, 0.15% formic acid in acetonitrile. In both cases, samples were then vortexed for 15 s and centrifuged at 10 000 rcf for 10 min at 4 ºC to pellet precipitated material. 200 μL of supernatant was than transferred to a 250μL polypropylene HPLC vial.

### ‘Days to Bud Break’ (DTBB) values

Given the geographical distance between the farms, differences in dates of production season were normalized by converting the Julian calendar day into a new ‘Days To Bud Break’ (DTBB) value. The date of bud break at the different sites was predicted with historical meteorological data from the nearest weather station provided by Climate Services of Canada at http://climate.weather.gc.ca/historical_data/search_historic_data_e.html with the model of Raulier and Bernier [22]. Briefly, beginning from Dec 1^st^ 2018, the model uses the number of days where the mean temperature is below 10 ºC to determine a ‘Threshold Temperature Sum’ (Sw), defined by Raulier and Bernier as:

$$S_{w}=S_{w150}\times e^{\alpha (dc-150)}$$ (1)

d_c_ = the cumulative number of days where the mean temperature is below 10 ºC.

S_w150_ = estimated parameter, set to 16.891[22]

α = estimated parameter, set to -0.0675 [22]

Sw_150_ and α were taken directly from the model of Raulier and Bernier [22] based on the observed date of leaf emergence in the native range of sugar maple.The predicted date of bud break occurs when the calculated S_w_ is equal to the observed temperature sum, defined by Raulier and Bernier as the cumulative positive temperature difference between the daily mean temperature and 10 ºC. The Julian calendar date when a sap sample was collected was then converted into a DTBB value by calculating the number of days until the predicted date of bud break at that specific site. In addition, the ‘Index of dormancy release’ (S_bb_) of N’guyen et al., based on the Raulier and Bernier model was attempted [6].

### Non-targeted analysis by high resolution LC-MS

All LC-MS data was acquired with a Thermo Q-Exactive Orbitrap^®^ mass spectrometer coupled to an Agilent 1290 HPLC system. The following conditions were used for heated electrospray ionization (HESI): capillary voltage 3.9 kV; capillary temperature, 400°C; sheath gas, 17 arbitrary units; auxiliary gas, 8 units; probe heater temperature, 450°C and S-Lens RF level, 45%. HILIC and reverse phase analysis were performed in both positive and negative ionization mode.

#### Non-polar analysis by C18 chromatography

For the reverse phase analysis, 5μL of sample was injected onto a Zorbax Eclipse Plus RRHD C18 column maintained at 35°C (2.1 × 50 mm, 1.8 μm; Agilent). Mobile phase A (0.1% formic acid in LC-MS grade H_2_O,Thermo Fisher Scientific) began at 100% and was held for 1.25 min. Mobile phase B (0.1% formic acid in LC-MS grade acetonitrile, Thermo Fisher Scientific) was then increased to 50% over 1.75 min, and 100% over 0.5 min. Mobile phase B was maintained at 100% for 1.5 min and returned to 0% over 0.5 min. The reverse phase samples were analyzed by a 140,000 resolution full MS analysis in the mass range of m/z 58–870. To facilitate identification of compounds, two composite samples, generated by mixing equal amounts of 20 samples each were analyzed by a top 3 data-dependent acquisition (DDA) experiment comprised of a full MS scan in the mass range of *m/z* 58–870 at 35,000 resolution, followed by MS/MS scans at 17,500 resolution, isolation window of m/z 1.2 and collision energy of 28.

#### Polar analysis by HILIC chromatography

For the HILIC analysis, 2 μL of sample was injected onto a Agilent HILIC-Z (2.1 × 100 mm, 2.7 μm; Agilent) column maintained at 35°C. Compounds were resolved with mobile phases of 20 mM ammonium formate in water (A) and 20 mM ammonium formate in 90% acetonitrile (B) operating with the following gradient: 0 min, 100% B; 0.5 min, 100% B; 5.3 min, 80% B; 9.5 min, 30% B; 13.5 min, 30% B, 14.5 min 100% B and 16.5 min, 100% B. The HILIC acquisition mode was a top 3 data-dependent acquisition (DDA) experiment comprised of a full MS scan in the mass range of m/z 58–870 at 35,000 resolution, followed by MS/MS scans at 17,500 resolution, isolation window of m/z 1.2 and collision energy of 28. The levels of amino acids and other polar metabolites was achieved also analyzing calibration solutions of these compounds under the same experimental conditions. Quantification was performed in full MS (±3 ppm) using Xcalibur Quantitative software package.

### LC-MS data analysis

Thermo.raw files were converted to.mzml format using Protewizard [23], with peak peaking filter applied. Features were detected using the XCMS package [24] with the centWave method (ppm tolerance 3.0) [25]. The signal to noise threshold was set to 5, noise was set to 5×10^5^ and pre-filter was set to five scans with a minimum 5,000 intensity. Retention time correction was conducted using the obiwarp method [26]. Grouping of features was set to those present in at least 0.1% of all samples (retention time deviation 5 s; *m/z* width, 0.015). The ‘fillPeaks’ function was used with default settings. Zero values were imputed by 2/3 the minimum peak area value of a specific feature across all samples. PCA plots were obtained by log transforming the imputed XCMS peak area values, and ‘pareto’ scaling in Rstudio. Volcano plots were also generated using the imputed XCMS peak area values. Compounds were identified by accurate mass, comparison of retention times to authentic standards or by accurate mass and also comparison of fragmentation patterns to MS/MS databases [27]. This data is available at the NIH Common Fund's National Metabolomics Data Repository (NMDR) website, the Metabolomics Workbench, https://www.metabolomicsworkbench.org where it has been assigned Project ID 1989.

## Results

### Days to Bud Break (DTBB)

**Fig 1:**
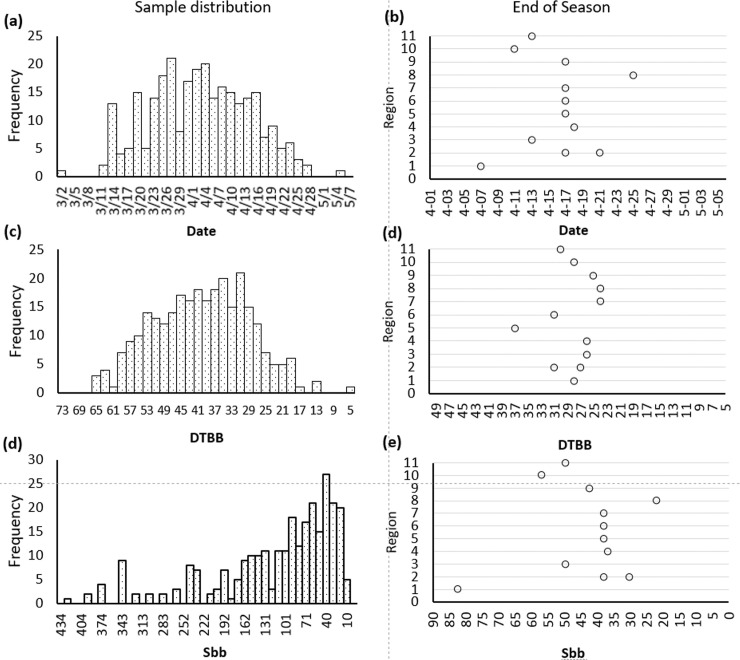
Distribution of sap samples collected from Ontario regions in the 2019 maple syrup production season. Frequency of samples are tallied by (a) Julian date, (c) DTBB, and (d) S_bb_. The last sap sample collected to make ‘good’ syrup as defined by producer surveys are plotted by region against (b) Julian Date, (d) DTBB and (e) S_bb_. Several producers continued collection of sap samples after production had ceased or off-flavors had been identified.

Throughout the 2019 maple syrup production season, 12 producers across all 11 provincial OMSPA regions collected 282 sap samples. Time of syrup production across the regions varied considerably due to the large geographic and climate differences. In an effort to normalize the sap collection season across the regions, the Julian dates were transformed to a ‘days to bud break’ (DTBB) value using the model of Raulier and Bernier [22]. Zero DTBB represents the predicted date of leaf emergence, calculated using meteorological data. The southernmost site, located in Region 1, was the first to end syrup production on April 7^th^, whereas the last sap collected for syrup occurred on April 25th in Region 8, a difference of 18 days. When the Julian dates were converted to DTBB, the final collection dates of Region 1 and Region 8 were narrowed to 28 and 24 DTBB, respectively (Fig 1B and 1D). Previously, differences in the dates of maple syrup production across a geographical range were addressed using a similar S_bb_ index proposed by N'guyen, also based on the Raulier and Bernier model [6, 22]. Application of the S_bb_ from the model of N'guyen et al. led to larger variations in in the sap collection season across Ontario (Fig 1E). The Sbb index values for the last days of syrup production for regions 1 and 8 by this method were 82 and 22, respectively. The S_bb_ index values for the last day of syrup production for regions 1 and 8 was 82 and 22 respectively. A normal distribution was obtained for the number of sap samples collected across the province by date (Fig 1A). When the frequency of samples collected is tallied by DTBB, the distribution becomes truncated, indicating a normalization of the season at the production sites has occurred (Fig 1C). In order to effectively compare the chemical composition of maple sap over the production season of all regions, the DTBB value in place of a traditional Julian date was used, and 'late' season sap is defined as samples collected at a DTBB of 30 or less.

### Non-targeted chemical analysis

**Fig 2:**
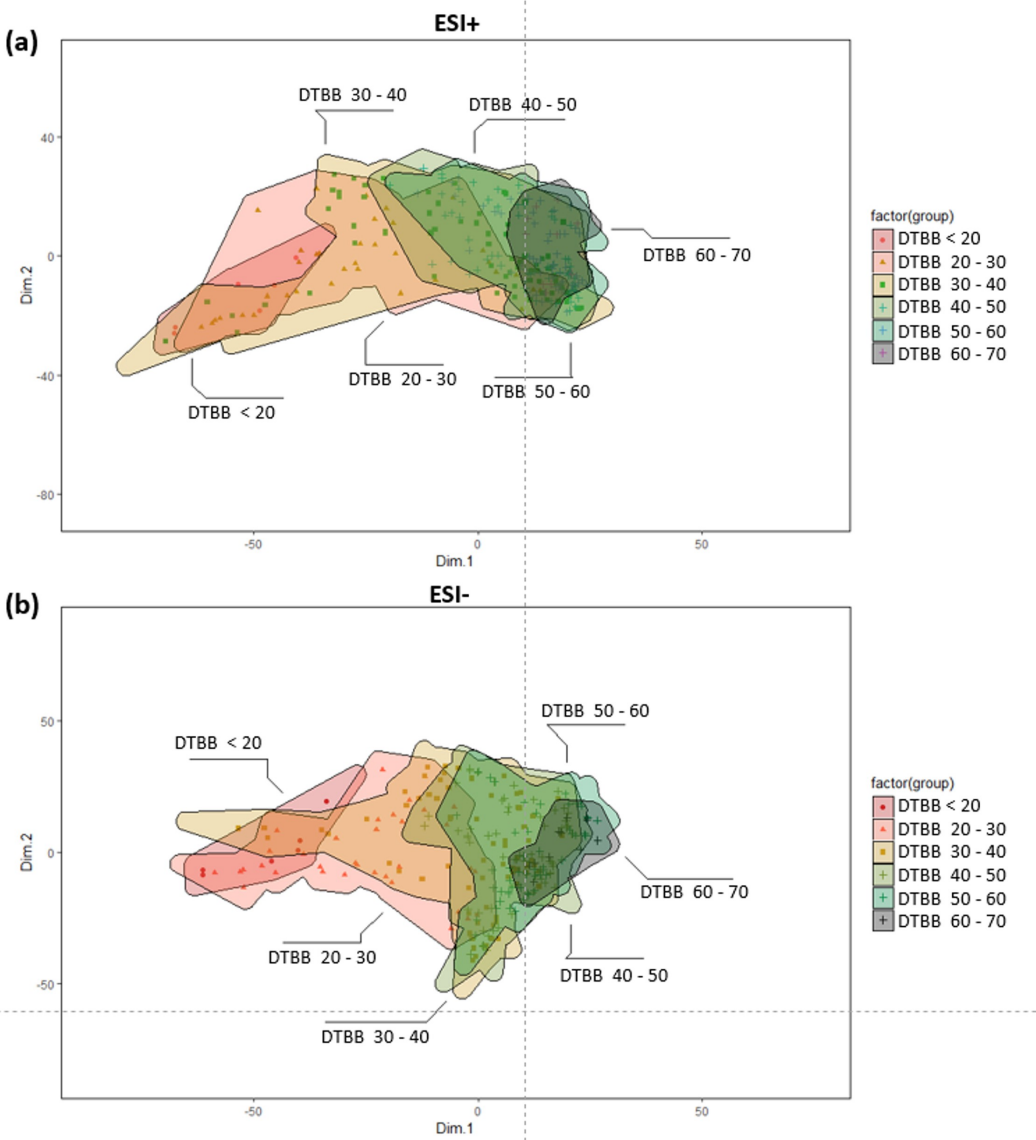
PCA plots of chemical features detected by C18 chromatography, high resolution mass spectrometry in (a) positive and (b) negative ESI ionization modes. Samples were organized by DTBB, showing co-clustering by DTBB > 40, followed by a shift with the later date samples.

**Fig 3:**
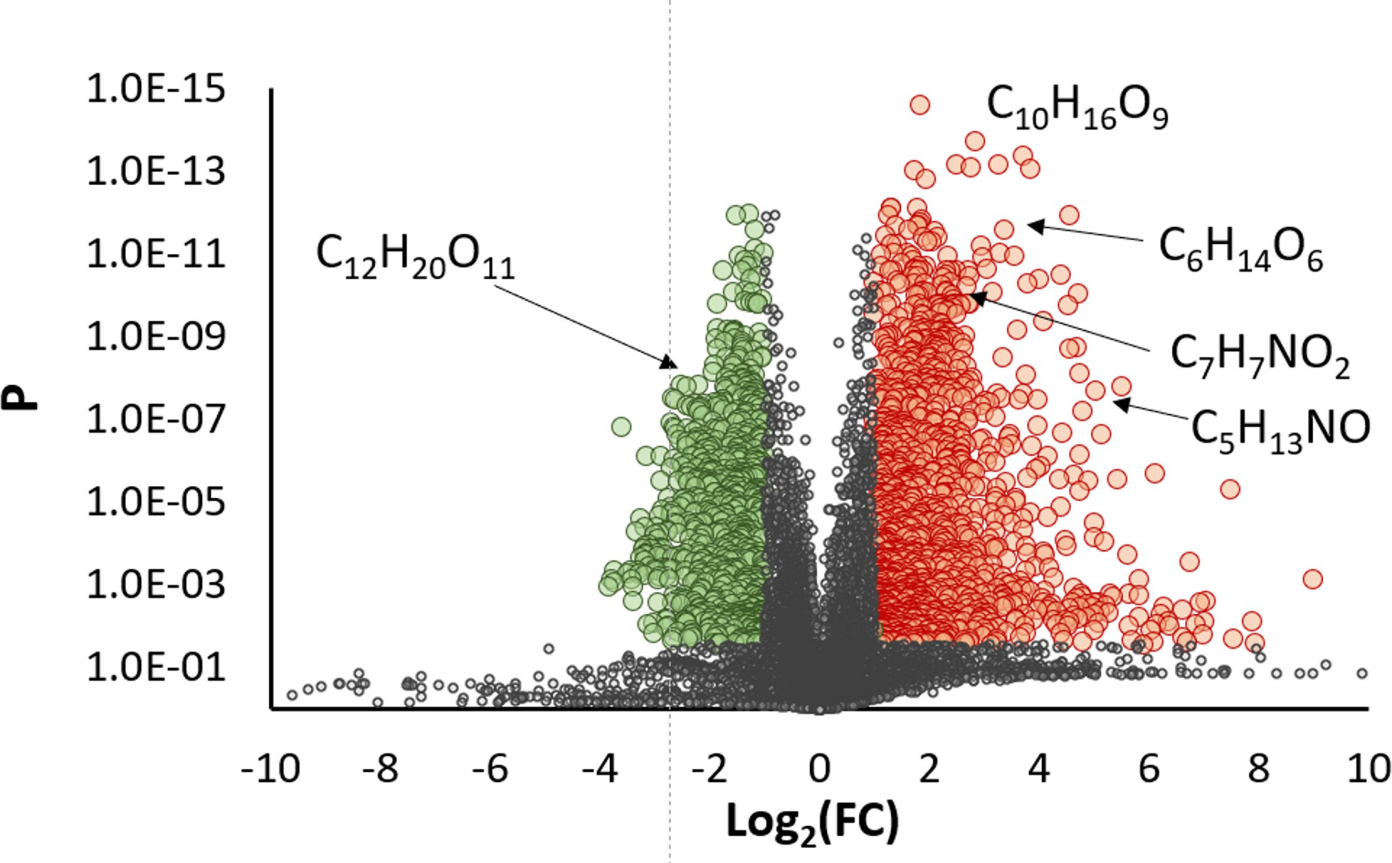
Volcano plot showing differentially present chemical features detected in positive ionization mode. False discovery rate (P) was controlled using the Benjamini-Hochburg procedure. Red circles represent features that are present in significantly increased amounts (P < 0.01; log_2_ > 1) in late season sap, while green circles are features that have significantly decreased (P < 0.01; log_2_ < -1).

The maple sap samples were analyzed by high resolution mass spectrometry in both positive and negative ionization. The XCMS software package extracted the peak areas of the chemical features within each sample and visualized by principal component analysis (Fig 2). In both positive and negative ionization modes, sap samples collected at 40 DTBB or earlier clustered together. Sap samples collected after 30 DTBB showed divergence from the main cluster of samples, indicating that alterations to the chemical composition occur most convincingly after this time. The principle features responsible for the observed differences of the early and late season samples in Fig 2, were determined by comparing their relative peak area in samples collected after 30 DTBB with those collected prior. In total, 1164 features significantly increased in the late season sample, while 678 significantly decreased (P < 0.01; log_2_ > |1|) in positive ionization mode (Fig 3). C_10_H_16_O_9_ was one of the most significantly increased compounds (P = 1.98× 10^−14^; log_2_ = 2.85) in late season sap. This compound was putatively identified by MS/MS as a succinyl-hexose, although the specific hexose (glucose, fructose) could not be determined.

**Fig 4:**
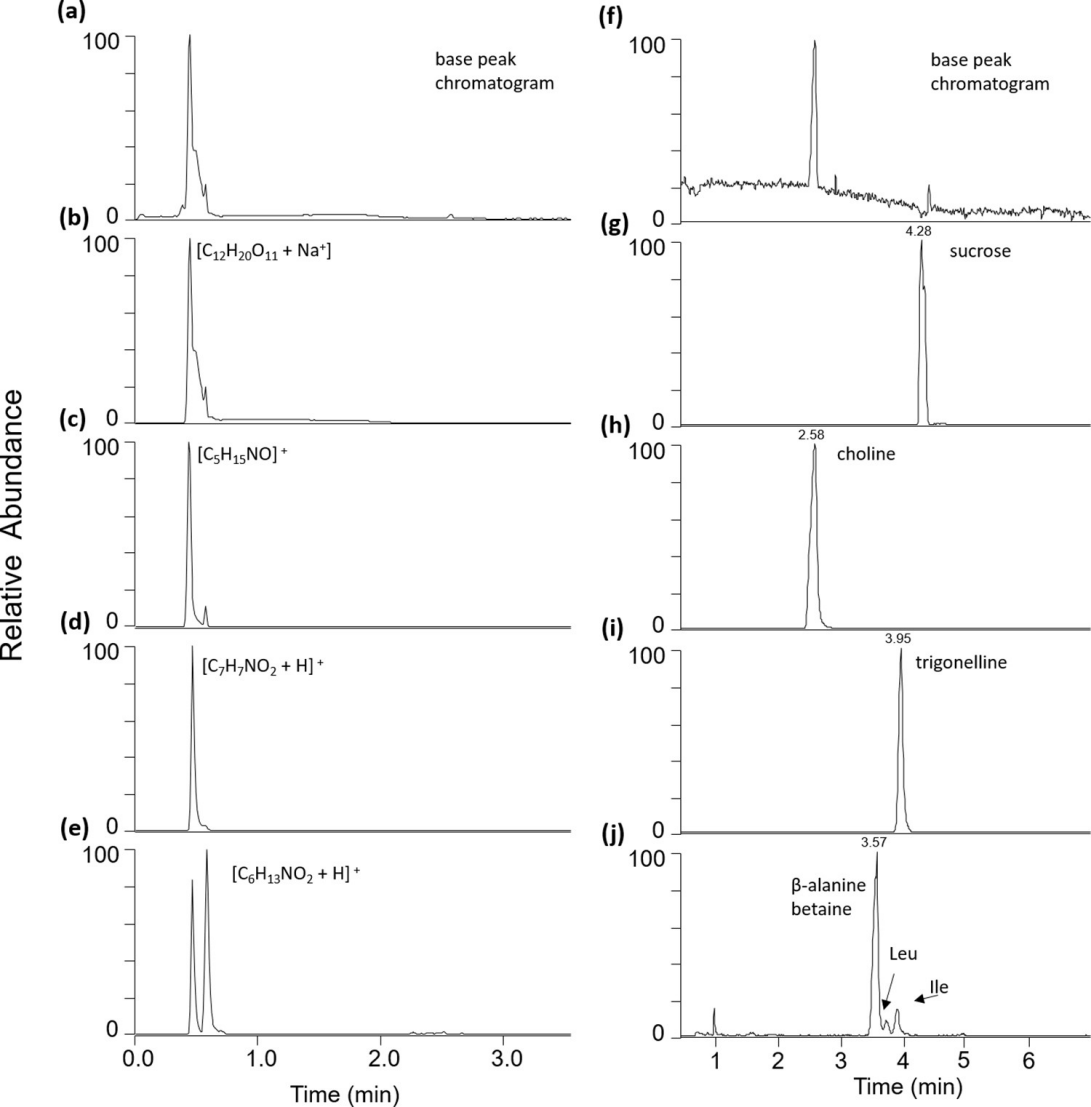
Samples were analyzed by (a-e) C18 chromatography (f-j) and HILIC chromatography. The (a) base peak chromatogram of C18 analyzed samples shows an abundance of polar compounds eluting in the void volume, including (b-e) significantly expressed components. To reliably determine these polar compounds, HILIC chromatography was used (f-j). The putative Leu, Ile amino acids identified in (e) C18, were determined to be (j) β-alanine-betaine.

When analyzed after C18 chromatography, the majority of compounds that were significantly different were polar and eluted in the void volume (Fig 4A). A disaccharide (C_12_H_20_O_11_) was significantly reduced in late season sap, whereas the number of putative amino acids, and other nitrogenated compounds of formula C_5_H_13_NO and C_7_H_7_NO_2_ were significantly increased. In order to confirm the identity of these differentially expressed polar compounds, HILIC chromatography was performed on a subset of 108 samples. As expected, the significantly decreased disaccharide was sucrose, whereas the C_5_H_14_NO and C_7_H_7_NO_2_ were choline and trigonelline, respectively (Fig 4G–4I). When analyzed by C18 chromatography, two features having the same formula as the amino acid leucine/isoleucine were also detected (Fig 4E). When analyzed by HILIC chromatography, minor amounts of leucine and isoleucine were found, and the primary compound of formula C_6_H_13_NO_2_ was β-alanine-betaine (Fig 4J).

### Saccharide analysis

**Fig 5:**
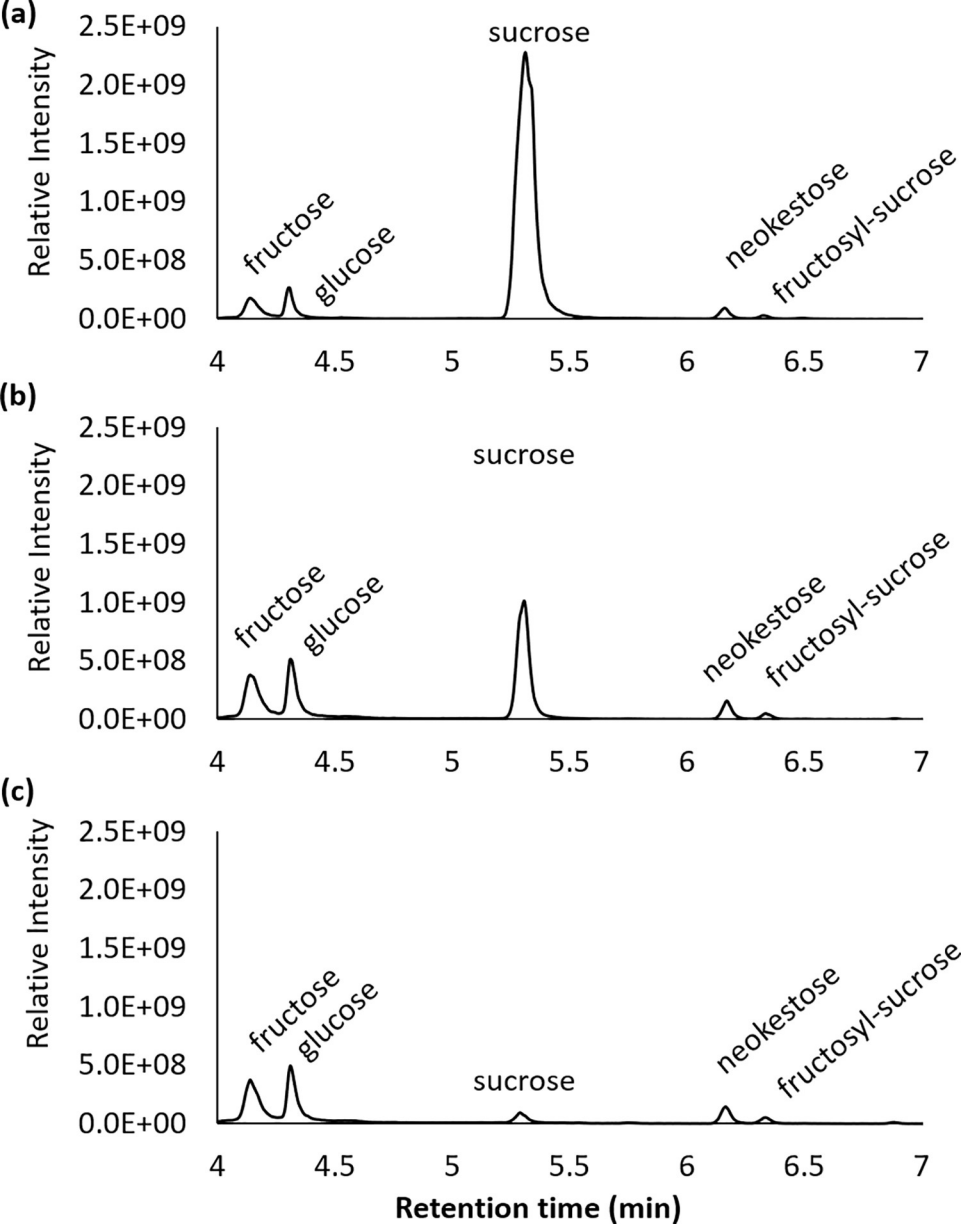
Extracted ion chromatograms of HILIC analyzed fructose, glucose, sucrose, neokestose, and fructosyl-sucrose, all detected as the [M-HCO2-] adduct in negative ionization mode. (a) In early season sap DTBB 50–60, sucrose was the major saccharide detected, decreased after (b) DTBB 30, and was present at low concentrations after (c) DTBB 20.

Sucrose, an important component of maple sap which was present in much lower concentrations in late spring than at the beginning of the sap run (Fig 3). Early season was defined as DTBB>60 and late spring season was DTBB < 30, and 108 samples whereas analyzed in closer detail by HILIC chromatography (Fig 5). In addition to sucrose, and the monosaccharides fructose and glucose, two major trisaccharides were also detected identified as neokestose and another fructosyl-sucrose were also detected. Two major trisaccharides identified as neokestose and fructosyl-sucrose were also detected (Fig 5).

**Fig 6:**
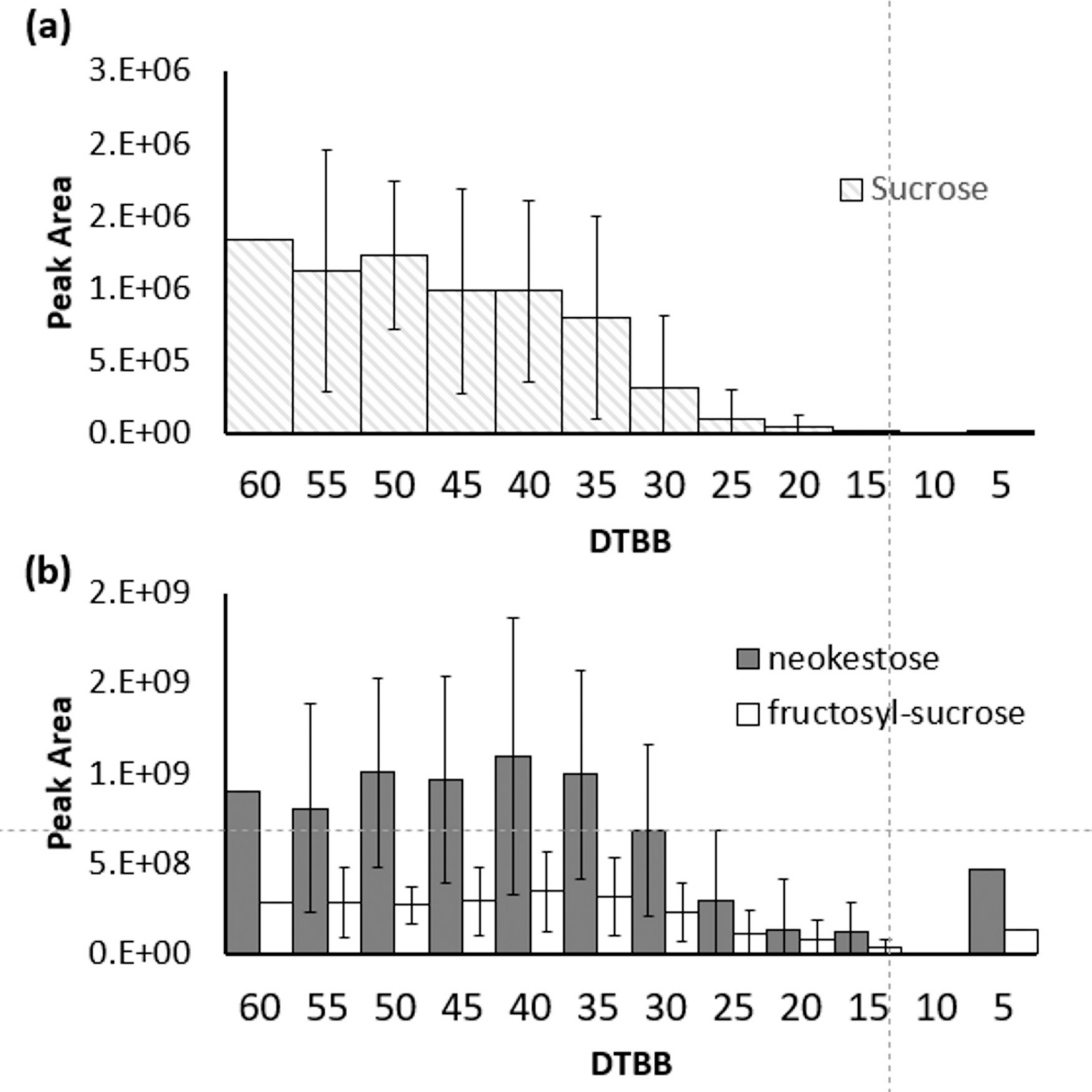
Average peaks areas of (a) sucrose and (b) the trisaccharides neokestose and fructosyl-sucrose. Samples were plotted in bins of 5 DTBB units across the 2019 season. Significant variations in the amounts of sucrose and trisaccharides present existed across the regions; however, the general trend showed relatively stable concentrations before 40 DTBB. Concentrations rapidly decreased towards the end of the production season.

Relative peak areas of sucrose showed significant variations across the different study sites. However, the pattern of change in the sap was similar from the beginning of the season to DTBB 40 (Fig 6A). After this point, the concentration of sucrose decreased rapidly and was only present at minor levels in samples of DTBB < 20 (Figs 5 and 6). The less studied trisaccharides neokestose and fructosyl-sucrose showed a similar trend as sucrose over the same collection period. Concentrations remained stable until DTBB 40 and decrease towards late season, with neokestose showing a marked decrease (Fig 6B).

### Amino acid analysis

**Fig 7:**
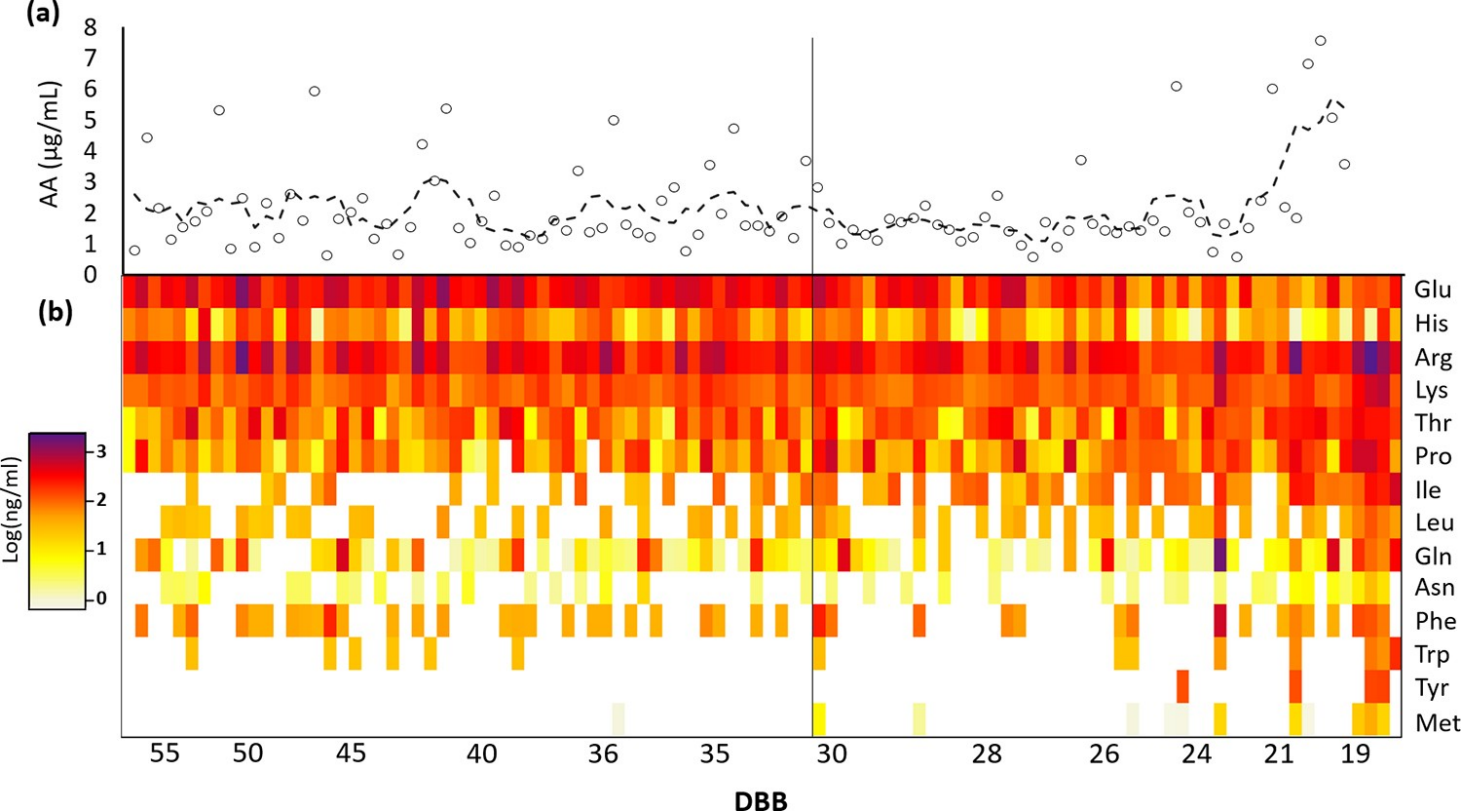
Heat map of target amino acids showing their (a) combined concentrations in μg/ml and (b) individual contributions. Glutamic acid and Histidine showed an overall decrease across the season, whereas the other amino acids showed a degree of increase. Methionine, tyrosine and tryptophan were only rarely detected in early season sap, however were detected frequently near the end of the season.

Many of the features that were notably altered between late and early season sap (Fig 3) were identified as proteogenic amino acids based on accurate mass. Since these compounds eluted in the void volume, their identities were confirmed and quantified by HILIC chromatography in a subset of samples (Fig 7). These targeted amino acids had combined concentrations of 1–2 μg/mL over the season; some samples had exceptionally high amounts of amino acids (Fig 7A). During the late season, the total concentration of the target amino acids showed a clear increase. Glutamic acid and histidine had a decreasing trend across the season, while the other amino acids had varying degrees of increase. Of note, leucine, isoleucine, lysine and glutamine, were stable throughout the season and increased considerably in the late season (Fig 7). Methionine, tyrosine and tryptophan were rarely detected in early season sap but were frequently identified in late season (Fig 7B).

A comparison of the dominant metabolites between the different sites revealed that methionine and asparagine tended towards greater concentrations (>5 ng/ml) towards the end of the season. In the final few samples, the ratio of the two approached 1:1 (S1 Table). In absolute terms, glutamine was greater than the related nitrogen rich amino acid asparagine. Several sap samples collected in the early and mid-points of the season contained detectable levels of glutamine.

### Other characteristics of late season sap

**Fig 8:**
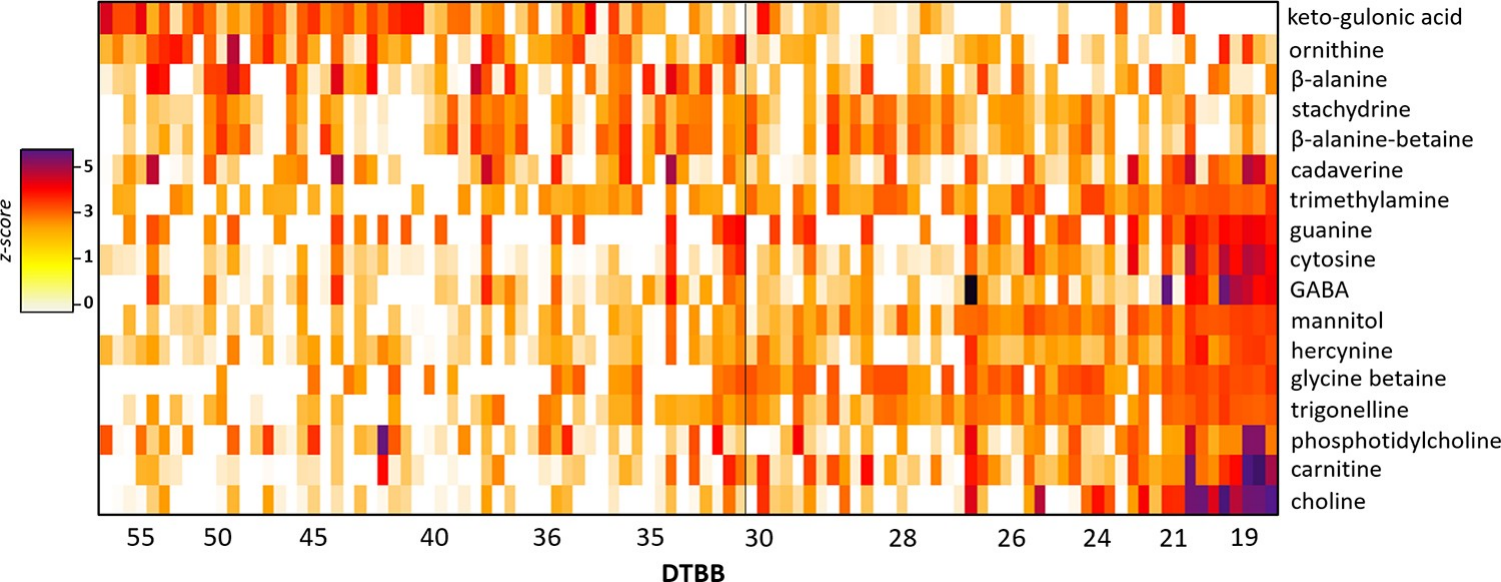
Heat map showing select differentially expressed compounds across the 2019 production season, measured by HILIC, high resolution MS. Peak areas were log transformed, and the compounds were normalised individually. Betaines were a major class of compounds that increased in late season sap.

**Fig 9:**
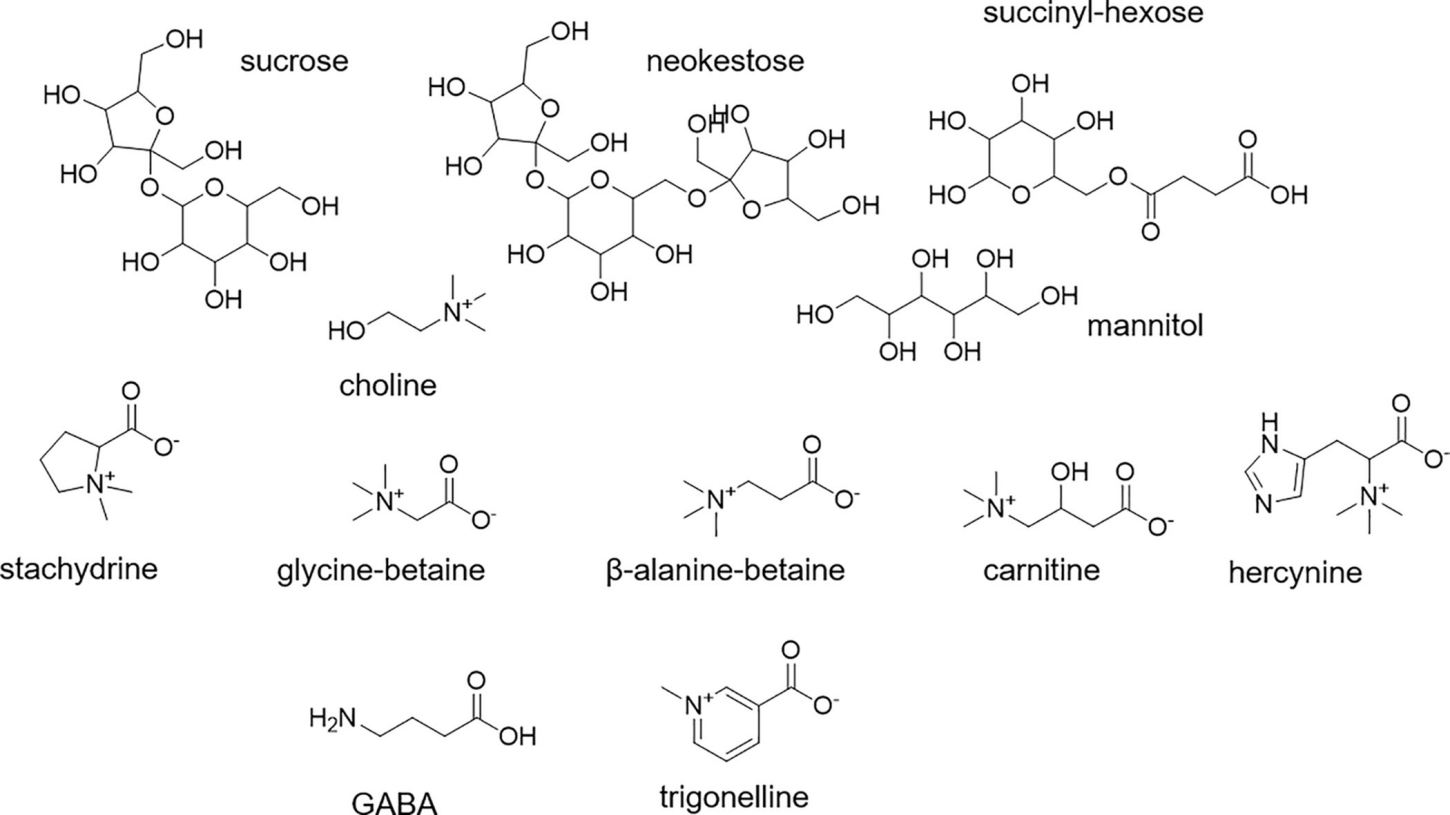
Chemical structures of select compounds that showed significant differences across the 2019 maple syrup production season.

**Fig 10:**
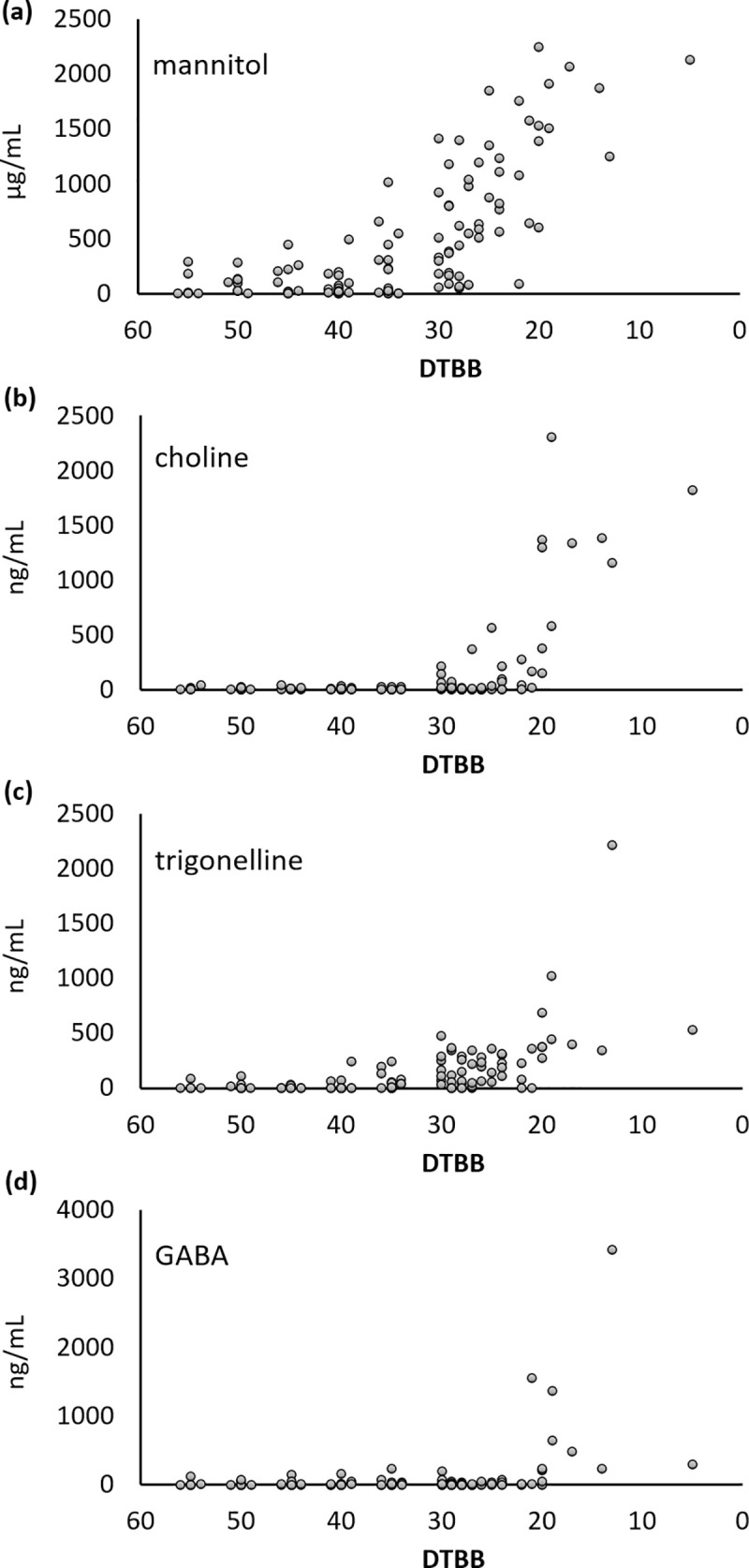
Concentrations of (a) mannitol, (b) choline, (c) trigonelline and (d) GABA in sap samples across the production season. Mannitol showed a site dependent gradual increase, while choline, trigonelline and GABA showed a sudden increase in the late season.

In addition to the amino acids, a series of other chemical features were found to be differentially expressed in early and late season sap (Fig 2). As many of these compounds eluted in the void volume, they were monitored by HILIC chromatography and identified by MS/MS [27] and/or comparison with commercial standards (Fig 8). Keto-gulonic acid, identified by comparing experimental MS/MS with database spectra [27], and ornithine showed a decrease in concentration across the season (Fig 8). The nucleotides guanine and cytosine, and amines cadaverine, and trimethylamine showed a significant increase in the late season sap, with pronounced increases occurring at DTBB < 20. A notable class of compounds that showed increases in the late season were hercynine, glycine betaine, phosphatidylcholine, carnitine and choline. These betaines and choline share a common quaternary ammonium moiety (Fig 9). The absolute concentrations of some of the prominent compounds that increased in late season sap were quantified (Fig 10). Mannitol was detected in most samples in the early season, at concentrations below or near 500 μg/ml and increased to over 2000 μg/ml near the late season. Although the trend of increasing concentrations of mannitol was true across all sites, a large, the timing of when the increase began varied by site.

Choline was present at non-detectable or trace levels up to 30 DTBB, after which, significant increases occurred at all sites. All samples collected after 20 DTBB had detectable levels of choline (Fig 10B). Another quaternary amine based compound, trigonelline (Fig 8), showed similar trends to choline; but increased in concentration earlier, at or near 30 DTBB. Finally, the non-proteogenic amino acid GABA had a concentration of >3mg/mL after 20 DTBB. This was significant, considering that the total of the other amino acids quantified were approximately 5μg/mL after 20 DTBB.

## Discussion

Though the chemical composition of maple syrup and to a lesser extent, maple sap has been investigated for decades, the complete suite of chemicals responsible for maple flavors have not been identified. Less is known about the chemical precursors in the sap that react during the heating/evaporation process to make these flavors. Although the precise sensory qualities of the syrup are greatly affected by how sap is processed, ‘buddy’ off-flavor is common across regions when late season sap is used. In an effort to find common chemicals that are present in saps across the Province of Ontario, particularly in the late season, it was necessary to normalize the sap collection times. The seasonal release sap from sugar maples results from positive stem pressures. This is initiated and sustained by freeze-thaw diurnal cycles. Thus, the specific climate of a maple syrup production site is the determining factor of when sap flows are amenable for collection. Lagacé et al. (2015) divided the production season into quartiles based on the total amount of syrup produced in a season. N’guyen introduced the S_bb_ index based on the predicted date of leaf emergence [22]. When we applied the S_bb_ index to this data set, we found that the end season production dates of some sites were moved further apart (Fig 1E). Additionally, the structure of the S_bb_ is not linear, meaning 1 day is not equal to 1 S_bb_ unit, and the distribution of samples collected was skewed to later S_bb_ indices (Fig 1D). The DTBB value, which is also based on the predicted date to leaf emergence, proved superior for normalizing the production seasons across all regions. Site specific forest genetics may have caused this, but it is more likely the result of the nearest meteorological measurement station not being representative of the conditions at the site. When all study sites are included, the end of the season occurred at a DTBB of 28.1 ± 3.7 days; however, when region 5 is excluded, this value narrows to 27.3 ± 2.6 days.

In previous studies of sugar maple sap, known chemicals were targeted for analysis. A drawback of the targeted approach is the limited opportunity for the new findings. In this work a metabolomic approach revealed that no substantial differences occurred in the sap collected at 40 DTBB or earlier. In contrast, significant chemical changes to the sap are observed at DTBB 30 or less. Thus in this study, we defined ‘late’ season sap as DTBB 30 or later. The non-targeted chemical analysis approach used in this study revealed that many of the significantly altered chemicals were polar and not retained by the reverse phase C18 column. Given that the maple sap is mostly water, it is not surprising that the majority the components reported are polar. One of the most significantly increased compounds was succinyl-hexose. The compound was putatively identified based on its appearance as a [M-H]^-^ molecular ion in negative ionisation mode as well as [M+Na]^+^ in positive mode and the distinctive neutral loss of the hexose moiety (-C_6_H_10_O_5_) in MS/MS in positive mode. To our knowledge, this compound has not been previously reported in sugar maple sap.

The decrease of sucrose content in maple sap in late season sap has previously been observed [21]. The causative agent of this decrease at the end of the maple production season appears to be the result of microbial activity. In 1947, Holgate reported that the sugar content diminished in the late season, conversely, when the sap was collected aseptically, the total sugar percent remained above 2% at the end of the season [8]. The sap samples were collected by the 12 producers in an identical manner to sap that would be processed into syrup and as such, were subject to microbial activity. Based on the decrease in sucrose, microbial activity accelerates after 30 DTBB (Fig 6). In the present work, the nucleobases guanine and cytosine were also found to increase in the late season samples. This may be due to transport to the swelling buds or from increased microbial activity in the sap.

We observed an increase of amino acids and other nitrogenous compounds in late season sap (Figs 7 and 8). The 1947 study noted above found that the nitrogen content of sap increased towards the end of the season regardless of sterility [8]. Recently, N’guyen and co-workers have proposed that the small sulphur-containing compounds, DMDS (C_2_H_6_S_2_) and DMTS (C_2_H_6_S_3_), may be responsible for the late season, ‘buddy’ off-flavor. In our analytical approach, cysteine could not be detected however, methionine was detected almost exclusively in the late season. At high temperatures, methionine decomposes mainly into methanethiol [28] via a Strecker degradation mechanism and can be readily oxidized aerobically to dimethyl disulfide [29].

The alditol, mannitol is widespread throughout the plant kingdom [30]. This polyol is an osmoregulator and is synthesized by plants in response to conditions that cause cellular dehydration [31]. Although the mannitol detected in this study might be biosynthesized by fungal sources [32], it may also be an indication of photosynthetic activity occurring within the tree [33–35]. Though leaves had not yet emerged when mannitol concentrations began to rise, stem photosynthesis is a crucial process occurring in woody plants [36]. Mannitol is likely not directly implicated in the development of the ‘buddy’ off-flavor in maple syrup, yet it may serve as a suitable bio-measure for determining a tree’s release from dormancy, and the potential for the off-flavored syrup to be produced.

Starch and sucrose are the most abundant reserve of carbohydrates in plants; however, fructans contribute around 15% of the total carbohydrates reserve [37, 38]. Fructans act as a biological protection factor against cold weather, stabilize the cell membrane and regulate osmotic pressure.[39] Neokestose has been characterized as a cell membrane protector in grain plants that accumulates in phloem and xylem tissue [40]. The unique characteristics of neokestose and fructans are their ability to stop the leaking of the soluble content in the liposome during freezing conditions [41]. Fructose oligomers and polymers can also be found attached to sucrose. Fructosyl-sucrose, a fructan which accumulates in vacuoles and was reported in sugar maple sap in 1961 [42].

An important finding of this work is that an entire class of quaternary ammonium bearing compounds, appeared in late season sap (Fig 8). The cells of many plants use these and similar compounds, known as ‘compatible solutes’ [43] to maintain turgor pressure and acclimatize plants to environmental stresses [44]. Like mannitol, the emergence of these compounds in the late season sap is likely a sign that the cellular activity within the tree is intensifying. Unlike mannitol, many of these compounds likely participate in the development of flavor compounds during the heating process. These quaternary ammonium containing compounds will not undergo traditional Maillard type reactions with reducing sugars, however, they have been shown to act as methylating agents [45]. Trigonelline for example can be degraded thermally and produce flavor components during the roasting process in coffee beans [46].

The classes of compounds which most likely contribute to the unfavorable, late season aftertaste in maple syrup are alkyl pyrazines and sulfides. Pyrazines such as those reported in late season or buddy sap have an aftertaste characterized as ‘malty’ and ‘astringent’ [47]. Related compounds are found in raw potatoes that have been stored for a long time. Compounds such as DMDS and DMTS are described as ‘peppery’ and ‘brassica’ flavours [48, 49]. As noted methionine and asparagine tended towards greater concentrations later in the season compared to early season values. Asparagine has been shown to be most efficiently converted to pyrazines compared to the other amino acids detected in sap [18]. In contrast, in foods, methionine is typically most important in producing the sulfides [50]. These two amino acids represent strong candidates for the development of poor after taste and thus targets for sap based *in situ* tests.

## Conclusions

The work presented developed a climate model based on the work done by Raulier and Bernier that normalized the weather data across the province of Ontario using the ‘Days To Bud Break’ count (DTBB). This resulted in a better characterization the maple sap samples in relation to climate, particularly towards the end of the season. Untargeted analysis based on principal component analysis was crucial to establish the DTBB 30 as the point where most significant chemical changes occurred. Later sap samples exhibited a decrease in sucrose concentrations and the increasing of reducing sugars glucose and fructose. Consistent with many previous studies, the nitrogen content was higher quantities in the late season samples DTBB< 30. The amino acids asparagine and methionine, both known precursors of off- flavours in food increased approximately considerably in late versus early season sap. One or both of these compounds might be useful markers for sap that will not be salable.

## Supporting information

S1 Table Concentrations isoleucine, methionine, glutamine, asparagine, choline, trigonelline and mannitol in *A*. *saccharum* sap. (DOCX)
