# Supplementary material for: Metabolomics reveals chemical changes in Acer saccharum sap over a maple syrup production season
Source: PLoS One. 2020 Aug 20;15(8):e0235787. doi: 10.1371/journal.pone.0235787 (PMC7444596; doi:10.1371/journal.pone.0235787)
Supplement: S1 Table — (DOCX) [file pone.0235787.s001.docx]

S1 Table

| Region | Date | DTBB | Isoleucine (ng/ml) | Methionine (ng / ml) | glutamine (ng/ml) | asparagine (ng/ml) | choline (ng/ml) | trigonelline (ng/ml) | mannitol (ug/ml) |
| --- | --- | --- | --- | --- | --- | --- | --- | --- | --- |
| Southwestern Ontario | 3/15/2019 | 51 | <MDL | <MDL | <MDL | 7 | <MDL | <MDL | <MDL |
|  | 3/16/2019 | 50 | 20 | <MDL | <MDL | <MDL | 1 | <MDL | 128 |
|  | 3/20/2019 | 46 | <MDL | <MDL | <MDL | 3 | 1.9 | <MDL | <MDL |
|  | 3/25/2019 | 41 | <MDL | <MDL | <MDL | 3 | 4 | <MDL | 40 |
|  | 3/30/2019 | 36 | <MDL | 0.847338 | 3 | <MDL | 1 | 195 | 308 |
|  | 4/5/2019 | 30 | <MDL | <MDL | 18 | <MDL | 5 | 291 | 507 |
|  | 4/6/2019 | 29 | <MDL | <MDL | 22 | 3.590165 | 8 | 369 | 804 |
| Grey Bruce & District A | 3/23/2019 | 56 | <MDL | <MDL | <MDL | <MDL | 2 | <MDL | 2 |
|  | 3/24/2019 | 55 | <MDL | <MDL | <MDL | 3 | 4 | <MDL | 6 |
|  | 4/2/2019 | 46 | <MDL | <MDL | <MDL | 3 | 36 | <MDL | 102 |
|  | 4/3/2019 | 45 | <MDL | <MDL | <MDL | 4 | 12 | 33 | 20 |
|  | 4/8/2019 | 40 | <MDL | <MDL | 3 | <MDL | 5 | <MDL | 166 |
|  | 4/13/2019 | 35 | <MDL | <MDL | 2 | <MDL | 2 | 246 | 307 |
|  | 4/18/2019 | 30 | <MDL | <MDL | 475 | <MDL | 143 | 106 | 181 |
|  | 4/21/2019 | 27 | <MDL | <MDL | 45 | <MDL | 365 | 341 | 1040 |
| Grey Bruce & District B | 3/24/2019 | 55 | <MDL | <MDL | 5 | 3 | 5 | <MDL | 286 |
|  | 3/29/2019 | 50 | <MDL | <MDL | 2 | <MDL | 1 | 29 | 279 |
|  | 4/3/2019 | 45 | <MDL | <MDL | 2 | <MDL | 5 | 20 | 445 |
|  | 4/7/2019 | 41 | <MDL | <MDL | <MDL | <MDL | 12 | 63 | 178 |
|  | 4/12/2019 | 36 | <MDL | <MDL | 5 | 6 | 28 | 130 | 659 |
| Waterloo & Wellington | 3/29/2019 | 41 | <MDL | <MDL | 110 | 2 | 3 | <MDL | 11 |
|  | 4/3/2019 | 36 | <MDL | <MDL | 2 | <MDL | 3 | <MDL | 7 |
|  | 4/4/2019 | 35 | <MDL | <MDL | <MDL | <MDL | 21 | <MDL | 21 |
|  | 4/10/2019 | 29 | <MDL | <MDL | <MDL | <MDL | 16 | <MDL | 168 |
|  | 4/13/2019 | 26 | <MDL | <MDL | 2 | <MDL | 19 | 231 | 1192 |
|  | 4/17/2019 | 22 | <MDL | <MDL | <MDL | 3 | 3 | <MDL | 83 |
| Simcoe & District | 3/20/2019 | 55 | <MDL | <MDL | 93 | <MDL | 4 | <MDL | 2 |
|  | 3/30/2019 | 45 | <MDL | <MDL | 20 | 2 | 5 | <MDL | 1 |
|  | 4/4/2019 | 40 | <MDL | <MDL | <MDL | 2 | 10 | <MDL | 17 |
|  | 4/9/2019 | 35 | 109 | <MDL | 2 | <MDL | 3 | 6 | 216 |
|  | 4/14/2019 | 30 | 101 | <MDL | 9 | 2 | 9 | 163 | 918 |
|  | 4/14/2019 | 30 | 91 | 8 | 15 | <MDL | 62 | 69 | 324 |
|  | 4/15/2019 | 29 | 94 | 2 | 14 | <MDL | 39 | 11 | 383 |
|  | 4/16/2019 | 28 | 21 | <MDL | <MDL | <MDL | 16 | <MDL | 617 |
|  | 4/18/2019 | 26 | 108 | <MDL | 312 | 2 | 2 | 195 | 634 |
|  | 4/20/2019 | 24 | 215 | 16 | 1757 | 3 | 2 | 222 | 766 |
|  | 4/24/2019 | 20 | 91 | <MDL | 508 | 6 | 147 | 272 | 599 |
|  | 4/25/2019 | 19 | 103 | 21 | 198 | 10 | 2302 | 1022 | 1508 |
| Algoma & District | 3/30/2019 | 55 | <MDL | <MDL | 55 | <MDL | 13 | 86 | 178 |
|  | 4/4/2019 | 50 | <MDL | <MDL | 159 | <MDL | 22 | <MDL | 132 |
|  | 4/9/2019 | 45 | <MDL | <MDL | 515 | 3 | 8 | 26 | 223 |
|  | 4/14/2019 | 40 | <MDL | <MDL | 180 | <MDL | 35 | 67 | 193 |
|  | 4/19/2019 | 35 | <MDL | <MDL | 23 | <MDL | 19 | 54 | 1012 |
|  | 4/26/2019 | 28 | 28 | <MDL | 2 | <MDL | 2 | 40 | 45 |
| Algonquin & District | 3/29/2019 | 50 | <MDL | <MDL | 3 | <MDL | 4 | 113 | 28 |
|  | 4/13/2019 | 35 | <MDL | <MDL | 5 | <MDL | 5 | 45 | 0 |
|  | 4/14/2019 | 34 | <MDL | <MDL | 10 | <MDL | 24 | 80 | 1 |
| Haliburton & Kawartha | 4/1/2019 | 40 | <MDL | <MDL | 34 | <MDL | 2 | <MDL | 73 |
|  | 4/6/2019 | 35 | <MDL | <MDL | 2 | <MDL | 2 | 43 | 230 |
|  | 4/12/2019 | 29 | <MDL | <MDL | <MDL | 2 | 2 | 54 | 370 |
|  | 4/13/2019 | 28 | <MDL | <MDL | 3 | 2 | 1 | 62 | 439 |
|  | 4/16/2019 | 25 | <MDL | <MDL | <MDL | <MDL | 2 | 144 | 876 |
|  | 4/17/2019 | 24 | <MDL | <MDL | 2 | <MDL | 94 | 185 | 1234 |
|  | 4/19/2019 | 22 | 59 | <MDL | <MDL | <MDL | 278 | 225 | 1079 |
|  | 4/20/2019 | 21 | 24 | <MDL | 8 | <MDL | 161 | 359 | 1579 |
|  | 4/21/2019 | 20 | 81 | <MDL | 8 | <MDL | 377 | 374 | 1386 |
|  | 4/22/2019 | 19 | 81 | <MDL | 2 | 3 | 576 | 446 | 1911 |
| Ottawa Valley District | 4/4/2019 | 45 | 107 | <MDL | 17 | <MDL | 3 | <MDL | 1 |
|  | 4/9/2019 | 40 | 26 | <MDL | 2 | <MDL | 5 | <MDL | 0 |
|  | 4/9/2019 | 40 | <MDL | <MDL | 2 | <MDL | 2 | <MDL | 1 |
|  | 4/10/2019 | 39 | 126 | <MDL | 12 | 2 | 1 | 244 | 5 |
|  | 4/14/2019 | 35 | 71 | <MDL | 2 | <MDL | 1 | <MDL | 0 |
|  | 4/15/2019 | 34 | 90 | <MDL | 13 | <MDL | 2 | 39 | 1 |
|  | 4/19/2019 | 30 | 104 | <MDL | 3 | 2 | 1 | 248 | 55 |
|  | 4/20/2019 | 29 | 140 | <MDL | 2 | <MDL | 1 | 341 | 188 |
|  | 4/21/2019 | 28 | 125 | <MDL | <MDL | <MDL | 1 | 292 | 157 |
|  | 4/21/2019 | 28 | 109 | <MDL | <MDL | <MDL | 0 | 257 | 60 |
|  | 4/22/2019 | 27 | 81 | <MDL | <MDL | <MDL | 1 | 222 | 78 |
|  | 4/23/2019 | 26 | 85 | <MDL | 4 | <MDL | 3 | 282 | 584 |
|  | 4/24/2019 | 25 | 119 | <MDL | 4 | <MDL | 562 | 360 | 1351 |
|  | 4/25/2019 | 24 | 74 | <MDL | 2 | <MDL | 211 | 306 | 821 |
|  | 4/25/2019 | 24 | 106 | 1 | 9 | 3 | 71 | 309 | 560 |
|  | 4/27/2019 | 22 | 61 | <MDL | 6 | <MDL | 38 | 78 | 1754 |
|  | 4/28/2019 | 21 | <MDL | <MDL | 6 | 3 | 19 | <MDL | 637 |
|  | 4/29/2019 | 20 | 248 | 1 | 5 | 8 | 1370 | 690 | 1525 |
| Quinte & District | 3/19/2019 | 54 | 29 | <MDL | 2 | 2 | 36 | <MDL | 2 |
|  | 3/29/2019 | 44 | <MDL | <MDL | 2 | <MDL | 2 | <MDL | 23 |
|  | 4/2/2019 | 40 | <MDL | <MDL | 3 | 5 | 1 | <MDL | 48 |
|  | 4/7/2019 | 35 | <MDL | <MDL | 79 | <MDL | 3 | <MDL | 27 |
|  | 4/13/2019 | 29 | 35 | <MDL | 3 | <MDL | 5 | 1 | 792 |
|  | 4/15/2019 | 27 | 41 | <MDL | 2 | <MDL | 3 | 18 | 550 |
|  | 4/16/2019 | 26 | 37 | <MDL | <MDL | <MDL | 3 | 62 | 504 |
|  | 4/17/2019 | 25 | 74 | 1 | 2 | 2 | 33 | 53 | 1848 |
|  | 4/18/2019 | 24 | 109 | 1 | 6 | 2 | 4 | 109 | 1109 |
|  | 4/22/2019 | 20 | 272 | 14 | 63 | 7 | 1299 | 377 | 2243 |
|  | 4/25/2019 | 17 | 292 | 41 | 99 | 28 | 1334 | 396 | 2070 |
|  | 4/28/2019 | 14 | 243 | 17 | 67 | 13 | 1385 | 347 | 1874 |
|  | 5/7/2019 | 5 | 560 | <MDL | 353 | <MDL | 1824 | 527 | 2126 |
| Lanark & District | 3/20/2019 | 50 | <MDL | <MDL | 109 | <MDL | 1 | <MDL | 97 |
|  | 3/26/2019 | 44 | <MDL | <MDL | 26 | <MDL | 18 | <MDL | 256 |
|  | 3/31/2019 | 39 | <MDL | <MDL | 1 | <MDL | 14 | <MDL | 489 |
|  | 4/4/2019 | 35 | 27 | <MDL | 219 | 17 | 6 | 44 | 444 |
|  | 4/5/2019 | 34 | 51 | <MDL | 226 | 2 | 4 | 43 | 546 |
|  | 4/10/2019 | 29 | 36 | <MDL | 8 | 2 | 68 | <MDL | 1180 |
|  | 4/11/2019 | 28 | 90 | <MDL | <MDL | <MDL | 12 | 147 | 1396 |
|  | 4/12/2019 | 27 | 102 | <MDL | 2 | <MDL | 6 | 45 | 979 |
| Eastern | 3/25/2019 | 49 | 47 | <MDL | <MDL | <MDL | 1 | <MDL | 4 |
|  | 3/29/2019 | 45 | 47 | <MDL | 15 | <MDL | 0 | <MDL | 13 |
|  | 4/3/2019 | 40 | 43 | <MDL | 1 | <MDL | 1 | 2 | 25 |
|  | 4/4/2019 | 39 | 37 | <MDL | <MDL | 2 | 2 | <MDL | 93 |
|  | 4/8/2019 | 35 | 25 | <MDL | 2 | <MDL | 1 | <MDL | 45 |
|  | 4/13/2019 | 30 | 27 | <MDL | 4 | <MDL | 14 | 32 | 296 |
